# Reported Childhood Abuse and Stress Reactivity in Psychosis: A Conceptual Replication and Exploration of Statistical Approaches

**DOI:** 10.3389/fpsyt.2018.00639

**Published:** 2018-11-30

**Authors:** Jonas Weijers, Wolfgang Viechtbauer, Elisabeth Eurelings-Bontekoe, Jean-Paul Selten

**Affiliations:** ^1^Department of Psychosis Research, Rivierduinen Institute for Mental Health Care, Leiden, Netherlands; ^2^Division 2: Mental Health, Department of Psychiatry and Neuropsychology, School of Mental Health and Neuroscience, Maastricht University, Maastricht, Netherlands; ^3^Department of Clinical Psychology, Leiden University, Leiden, Netherlands

**Keywords:** experience sampling method, psychosis, childhood abuse, stress reactivity, non-normally distributed data

## Abstract

A previous study observed that reported childhood abuse moderated psychotic and emotional reactivity to stress among patients with non-affective psychotic disorder. However, that study used a type of analysis unsuited for skewed data. This study aimed (1) to replicate the study and (2) to examine whether we would obtain similar results using a statistical approach better suited to skewed data. Fifty-nine patients with non-affective psychotic disorder were examined for up to 6 days using an intensive diary method to assess levels of negative affect, psychosis, and daily-life stress. A mixed-linear regression largely replicated earlier findings, but a two-component analysis failed to replicate the moderating effect of reported childhood abuse. These results illustrate the importance of exploring different statistical approaches to skewed data. They may also indicate that stress sensitization does not offer a complete account for the effect of reported childhood abuse on psychotic symptom severity.

## Introduction

A growing body of evidence indicates that childhood abuse plays a role in the etiology of non-affective psychotic disorder (NAPD) (1–4). It has been suggested that an increased reactivity to stressful situations accounts for this (5–7). According to this view, cumulative exposure to traumatic experiences during childhood results in “behavioral sensitization,” a mechanism whereby previous exposure to adversity or stress renders individuals more sensitive to stress later in life, which in turn is thought to increase the risk for psychosis (8–10). Indeed, using the “experience sampling method” (ESM), it was shown that daily life stress is associated with the intensity of psychotic experiences in patients with NAPD (8). ESM is especially useful for examining dynamic processes in daily life, because it involves prompting participants to report on their experiences multiple times per day for an extended period of time. In this way, ESM constitutes an ecologically valid approach for the measurement of pathological experiences and stressors in daily life.

A number of ESM studies have shown that the severity of reported childhood abuse (RCA) can indeed affect the intensity of emotional and psychotic reactions to daily life stressors (11–15). RCA was shown to be correlated with greater negative emotional reactions or psychotic reactions to stress in nonclinical samples (11–13) and, with regard to NAPD, Lardinois et al. (14) showed that patients with high levels of RCA reported increased psychotic and emotional reactivity to event stress (i.e., when an unpleasant event happens) and activity stress (i.e., when an activity's difficulty exceeds one's capability). Similar observations were made by Reininghaus et al. (15), but they involved different types of stress, namely social stress (i.e., the unpleasantness of the participant's current social situation) and area-related stress (i.e., the unpleasantness of the participant's current neighborhood). Contrary to Lardinois et al. (14), however, Reininghaus et al. (15) did not find evidence that the relationship between event stress and negative affect or psychotic experiences was moderated by RCA.

Thus, the previous studies are in overall support of the hypothesis of behavioral sensitization to stress due to childhood abuse. However, it remains uncertain whether RCA specifically moderates the relationship between event stress and negative affect or psychotic experiences, as the findings of Reininghaus et al. (15) and Lardinois et al. (14) are in contradiction. Additionally, the finding by Lardinois et al. (14) concerning activity stress has not been replicated in an NAPD sample. In our view, this is important, since scientific claims can only be given credence in accordance with the reproducibility of their supporting evidence (16). Lastly, and most importantly, all studies concerning RCA and stress reactivity so far have employed mixed-effects linear regression models. Concerns may be raised about these analyses, since the outcome variables (i.e., negative affect and psychotic experiences) are rarely normally distributed and tend to be right-skewed (17), possibly violating normality assumptions underlying the models (with respect to the residuals and random effects). In light of this, mixed-effects linear regression analyses may not be suitable (17).

The current study sought to replicate and improve upon the study by Lardinois et al. (14) in several ways. First and foremost, we explored two statistical approaches for the analysis of the data: next to a mixed-linear regression, which mirrors the original study, we added a secondary analysis more suitable for the analysis of skewed outcomes to examine the sensitivity of the conclusions to the modeling approach used. Second, we used a semi-structured, observer-rated instrument to measure RCA, as opposed to the self-report questionnaire used in the previous study. This offered the opportunity to get an in-depth account of childhood experiences, while simultaneously providing a less subjective rating of the severity of abuse. Cristobal-Navaez et al. (12) suggest that interviews relying on objective definitions of adversity like the “Childhood Experience of Care and Abuse” (CECA) instrument allow for a more precise assessment. Third, the current study treated RCA as a continuous variable as opposed to the earlier studies that dichotomized RCA using a somewhat arbitrary cut-off point [e.g., (13–15)]. Many authors have argued against the artificial dichotomization of variables, as this can lead to a loss of information and power, lower reliability, inflated type I and type II errors, and potential bias (18–20). Fourth, some authors (21) have cast doubt on the reliability of the pen-and-paper experience sampling method used in the study by Lardinois and colleagues, preferring the use of electronic devices like the one used in the current study.

Specifically, the aim of this study is to test whether: (1) activity stress and event stress are related to negative affect and psychotic experiences and (2) whether the severity of RCA moderates the emotional and psychotic reactivity to activity stress and event stress.

## Materials and methods

### Sample and procedure

This study is part of a larger one (22) for which 90 patients with NAPD were recruited from community treatment teams at regional mental health care institutes in the Netherlands (the Rivierduinen Institute for Mental Health Care located in Leiden, Voorhout, and Zoetermeer, and the Altrecht Institute for Mental Health Care in Zeist). Diagnoses were established by psychiatrists prior to participation and included schizophrenia, schizoaffective disorder, schizophreniform disorder, brief psychotic disorder, delusional disorder, or psychotic disorder not otherwise specified. Their diagnosis was confirmed by researchers using the Comprehensive Assessment of Symptoms and History [CASH; (23)]. Only patients with a clinician diagnosis in the NAPD spectrum, were interviewed with the CASH for eligibility. Using the CASH it was revealed that all of the eligible participants who had been given a clinician diagnosis in the NAPD spectrum, also had NAPD according to the CASH. However, specific DSM-IV diagnoses (e.g., schizophrenia or schizo-affective disorder) could differ between clinicians and researchers. A reliability analysis revealed that there was an acceptable rate of agreement between clinicians and researchers with regard to specific NAPD diagnoses (Cronbach's alpha = 0.713). Further inclusion criteria were: (1) at least 18 years of age, (2) fluent command of the Dutch language, and (3) the start of their treatment for psychotic disorder had to be between 6 months and 10 years ago. Exclusion criteria were (1) intellectual disability, (2) illiteracy, or (3) substance addiction that necessitates detoxification. Treatment staff scanned their caseloads for possibly eligible patients. The researchers contacted these patients by telephone and invited them for an information consult and screening for in- and exclusion criteria.

### Measures

The current study used an intensive diary method almost identical to the original study. Like the study by Lardinois et al., participants were prompted by a signal (beep) 10 times a day to fill out a questionnaire for a total of 6 days. Please refer to the original study for more detailed information about this method (14). The current study only diverged from the previous one by using an electronic diary device (called the “PsyMate”) to collect the responses to the questions (instead of digital wristwatches and paper booklets).

As in previous studies [e.g., (11, 14)], stress reactivity was conceptualized as an increase in negative affect or psychotic experiences due to two types of stressful situations: activity stress and event stress. Activity stress was assessed by three items concerning the activity participants were engaging in when the beep was emitted. In particular, participants rated how unskilled they felt at the current activity (“I am not skilled to do this activity”), how difficult their current activity was (“This activity is difficult”), and to what degree they would rather engage in some other activity (“I would rather do something else”). The average of the three items was used as an indicator of activity stress. Event stress was assessed by a single item, asking participants to rate the pleasantness of the most important event preceding the current beep. The item was rated on a −3 (very unpleasant) to 3 (very pleasant) scale and was reverse scored before the analysis. Negative affect was assessed at each beep based on the average score of fives items: feeling insecure, lonely, guilty, down, and anxious. Psychotic experiences comprised the average score of seven items: feeling unreal, feeling disliked, feeling suspicious, fearing that one might be hurt by others, fearing to lose control, hearing voices, and experiencing that one's thoughts are being influenced by others. All of these items were rated on 7-point Likert scales.

For the ESM items measuring activity stress (3 items), negative affect (5 items), and psychotic experiences (7 items), we computed Cronbach's alpha values for the person-level means and the within-person mean centered values (to obtain information about the reliability of the items at the person and at the beep level). For activity stress, this yielded values of α = 0.71 and α = 0.46 for the person and beep levels, respectively. For negative affect, we found α = 0.93 and α = 0.69, respectively. Finally, for psychotic experiences, we obtained α = 0.91 and α = 0.70.

The CECA instrument was used to assess RCA. The CECA is a retrospective, semi-structured interview that allows researchers to rate the severity of possible abuse at age 0–16 years based on patients' accounts. The CECA has been found to be reliable and valid (24). A later study corroborated the validity of the CECA by comparing sisters' accounts of parental abuse, finding overall strong agreement between those accounts, even when only one sister had experienced the form of abuse reported (25).

Childhood abuse comprised four types of maltreatment: psychological abuse, physical abuse, sexual abuse, and parental conflict. Psychological abuse comprised extreme criticism, rejection, humiliation, or terrorizing. Physical abuse was defined as bodily harm that resulted in at least bruising. Sexual abuse was defined as the participant's report of any unwanted sexual incident, including sexual assault. Parental conflict was defined as the amount of fighting between the caregivers and/or the child. Each type of abuse was scored for both frequency and intensity. Frequency of abuse was rated on a five-point Likert-scale: 0 (never), 1 (rarely: once or twice), 2 (incidentally: more than two times, but not monthly), 3 (regularly: monthly or more often), or 4 (often: weekly or more often). Intensity of abuse was rated on a four-point Likert scale: 0 (none), 1 (some), 2 (moderate), or 3 (marked), with the exception of parental conflict, which was scored on a five-point scale that also included 4 (violence). The severity score for each type of abuse was the product of intensity and frequency. The total score, ranging from 0 to 52, was the sum of the severity scores for each subtype of abuse. For the analyses, we rescaled the variable by dividing the total score by 5 to avoid overly small coefficients. Hence, a one-unit increase on the rescaled RCA variable represents a 5-point increase on the original CECA total score.

### Data analysis

As in the previous study, the data have a multilevel structure: multiple observations (level 1) are nested within subjects (level 2). Generally, observations within a subject are related and hence the residuals are not independent and the assumption of statistical independence required for regular regression analyses is violated. Hierarchical linear modeling is therefore required (11, 26). Mixed-effects (multilevel) linear regression models were used to test the hypothesis that event stress and activity stress are related to negative affect and psychotic experiences. Intercepts and slopes for the stress variable were allowed to vary across subjects, with an unstructured variance-covariance matrix for the random effects. Next, we fitted models that included the stress variable, RCA, and their interaction to test whether RCA moderates stress reactivity to daily life stress.

The modeling approach described above constitutes the primary analysis, which is similar to the one carried out by Lardinois and colleagues (14). This analysis only deviated from the original one by not dichotomizing the RCA variable and allowing slopes to vary across subjects (only random intercepts were used in the original study; personal communication). Adding random slopes to the model is important to avoid inflated Type I error rates and overly narrow confidence intervals (27, 28).

With regard to the secondary analysis, examination of the data distribution indicated that a gamma distribution could provide an adequate fit to the data, but only when considering the subset of assessments where negative affect or psychotic experiences were actually present (i.e., when outcome >1). Therefore we decided to carry out a secondary analysis using a two-part or mixed-distribution model [e.g., see (29, 30)]. After rescaling the outcomes (i.e., negative affect and psychotic experiences) to a 0 to 6 range (i.e., y = outcome – 1), we conducted a mixed-effects logistic regression model for the presence vs. absence of any negative affect / psychotic experience (i.e., *y* > 0 vs. *y* = 0) and a mixed-effects gamma regression model with a log link for the intensity of negative affect/psychotic experiences when negative affect/psychotic experiences were actually present (i.e., if *y* > 0). The two-part models were fitted using maximum likelihood estimation with Gauss-Hermite quadrature (either with five or seven integration points). Correlated random intercepts and slopes were included for each component, but correlations across components (e.g., for the random intercepts of the logistic and gamma parts) were not allowed due to model convergence problems. The two-part model provides insights into two psychological mechanisms: How stress (and its interaction with RCA) is related to exhibiting any negative affect/psychotic experiences and how stress (and its interaction with RCA) is related to the intensity of negative affect/psychotic experiences when these experiences are actually present. Results are reported in terms of the estimated coefficients and corresponding 95% confidence intervals (CIs) and *p*-values. The fit of the standard mixed-effects model and the two-part model was compared by computing the difference in AIC values. Analyses were carried out with SPSS 23, R 3.4.0 using the “nlme” (31) and “lme4” (32) packages, and SAS/STAT software 9.2, using the “proc glimmix” and “proc nlmixed” procedures.

Lastly, we examined potential violations of statistical assumptions specific to mixed-effects models, namely the assumption of homoscedasticity and multicollinearity. Firstly, the assumption of homoscedasticity is relevant for any kind of regression analysis. Like the standard mixed-effects model used by Lardinois et al. (14), the assumption of homoscedasticity of residuals of the mixed-effects model used in the current study, was highly likely to be violated due to the bounded nature of the outcome variables (negative affect and psychotic experiences). An examination of a plot of the fitted values against the Pearson residuals for the current standard mixed-effects model, shows that this is clearly the case (lower variability for low fitted values, higher variability for higher fitted values). On the other hand, in the two-part model, we do not assume homoscedasticity to begin with, since the logistic and gamma parts of the model do not make such an assumption. Secondly, multicollinearity is also an issue for any kind of regression analysis. In the present case, the only predictors are the stress and the childhood abuse (CECA) variables. We examined the correlations between the subject-level means of the activity and event stress and the CECA variables. The correlations were equal to 0.27 and 0.42, respectively, which were not viewed to be particularly worrisome.

## Results

### Sample characteristics

Of the 90 patients who entered the study, nine participants refused to fill out any PsyMate questionnaire. One participant refused to answer questions concerning sexual abuse and was excluded. Lastly, 21 patients failed to fill out at least 20 questionnaires and as in previous studies (14, 16) were excluded from the analyses. The final sample used for the analyses therefore included 59 participants, providing responses to a total of 1926 beeps (on average, 32.6 responses per subject; *SD* = 8.6; range: 20–58). Demographic variables as well as the mean scores of the dependent and independent variables of the sample are shown in Table 1.

**Table 1:** Demographic and clinical characteristics of 59 patients with non-affective psychotic disorder who completed electronic diaries and reported on stress reactivity.

Age [Mean (SD), range]	31.83 (9.40), 19–57
Sex [M/F (%)]	34/25 (57.6%/42.4%)
CASH DSM-IV diagnosis [n (%)]	
Schizophrenia	34 (57.6%)
Psychotic Disorder NOS	10 (16.9%)
Schizoaffective Disorder	10 (16.9%)
Brief Psychotic Disorder	3 (5.1%)
Delusional Disorder	2 (3.4%)
	**Mean (SD), range**
Years since onset of psychotic disorder	5.61 (4.07), 1–22
Childhood abuse	8.88 (10.85), 0–43
**ESM variables:**	**Mean (SD), range**	**(Nonmissing) observations**
Negative Affect	2.17 (1.16), 1-7	1908
Psychotic Experiences	1.82 (1.00), 1-7	1893
Event stress	3.25 (1.43), 1-7	1878
Activity stress	2.80 (1.25), 1-7	1891

*CASH, Comprehensive Assessment of Symptoms and History; CECA, Childhood Experience of Care and Abuse instrument; ESM, Experience Sampling Method*.

### Primary analyses

#### Main effects

Both activity stress (*b* = 0.14, 95% CI [0.08, 0.20], *p* < 0.001) and event stress (*b* = 0.08, 95% CI [0.03, 0.12], *p* = 0.001) were positively and significantly associated with negative affect. Activity stress (*b* = 0.10, 95% CI [0.06, 0.14], *p* < 0.001) was also associated with the intensity of psychotic experiences, while event stress just failed to be significant (*b* = 0.03, 95% CI [−0.00, 0.05], *p* = 0.06).

#### Reported childhood abuse and emotional stress reactivity

Two analyses were conducted with negative affect as the outcome variable. In the first analysis, RCA, activity stress, and their interaction were added as predictors. The second analysis substituted activity stress for event stress. Results showed that RCA significantly moderated the relationship between activity stress and negative affect (*b* = 0.03, 95% CI [0.01, 0.06], *p* = 0.02), but not the relationship between event stress and negative affect (*b* = 0.02, 95% CI [0.00, 0.03], *p* = 0.07).

#### Reported childhood abuse and psychotic stress reactivity

Two additional analyses similar to the ones above were conducted with psychotic experiences as the outcome variable. Results showed that RCA significantly moderated the association between activity stress and psychotic experiences (*b* = 0.02, 95% CI [0.00, 0.04], *p* = 0.03), and between event stress and psychotic experiences (*b* = 0.01, 95% CI [0.00, 0.02], *p* = 0.03).

### Secondary analyses

The secondary analyses using the two-part models indicated that neither activity nor event stress was significantly related to the chance of the participant reporting any negative affect (i.e., any score above 0); neither was event stress related to the chance of the participant reporting any psychotic experience (all *p*'s ≥ 0.20; see Table 2 for full results). However, activity stress was related to the chance of the participant reporting any psychotic experience (*b* = 0.39, 95% CI [0.09, 0.70], *p* = 0.01). RCA did not significantly moderate the relationships between activity stress or event stress, and the presence of any negative affect or psychotic experience (all *p*'s ≥ 0.12; see Table 2 for full results).

**Table 2:** Results of the two-part analysis of main and interaction effects on negative affect and psychotic experiences.

**Independent variable or interaction**	**Dependent variable**	**Estimate**	**CI (95%)**	***P*-value**
activity stress	Any negative affect (i.e., *y* = 0 vs. *y >* 0)	0.60	−0.33 to 1.53	0.20
event stress	Any negative affect (i.e., *y* = 0 vs. *y >* 0)	−0.00	−0.25 to 0.24	0.98
activity stress	Any psychotic experience (i.e., *y =* 0 vs. *y >* 0)	0.39	0.09 to 0.70	0.01
event stress	Any psychotic experience (i.e., *y =* 0 vs. *y >* 0)	−0.01	−0.20 to 0.19	0.95
activity stress	Intensity of negative affect when present (i.e., if *y* > 0)	0.12	0.07 to 0.17	< 0.01
event stress	Intensity of negative affect when present (i.e., if *y* > 0)	0.06	0.03 to 0.10	< 0.01
activity stress	Intensity of psychotic experience when present (i.e., if *y* > 0)	0.12	0.06 to 0.18	< 0.01
event stress	Intensity of psychotic experience when present (i.e., if *y* > 0)	0.06	0.02 to 0.12	0.01
RCA*activity stress	Any negative affect (i.e., *y =* 0 vs. *y >* 0)	0.14	−0.03 to 0.30	0.12
RCA*event stress	Any negative affect (i.e., *y =* 0 vs. *y >* 0)	0.04	−0.09 to 0.17	0.57
RCA*activity stress	Any psychotic experience (i.e., *y =* 0 vs. *y >* 0)	0.07	−0.04 to 0.19	0.21
RCA*event stress	Any psychotic experience (i.e., *y =* 0 vs. *y >* 0)	0.00	−0.06 to 0.07	0.89
RCA*activity stress	Intensity of negative affect when present (i.e., if *y* > 0)	0.02	−0.01 to 0.04	0.22
RCA*event stress	Intensity of negative affect when present (i.e., if *y* > 0)	0.01	−0.01 to 0.03	0.36
RCA*activity stress	Intensity of psychotic experience when present (i.e., if *y* > 0)	0.01	−0.02 to 0.03	0.59
RCA*event stress	Intensity of psychotic experience when present (i.e., if *y* > 0)	0.00	−0.01 to 0.02	0.61

*RCA, Reported Childhood Abuse*.

On the other hand, both activity and event stress were significantly and positively related to the intensity of negative affect and psychotic experiences if present (all *p*'s ≤ 0.01; see Table 2 for full results). However, there was no evidence to suggest that RCA moderated the relationships between activity stress / event stress and the intensity of negative affect/psychotic experiences if present (all *p'*s ≥ 0.22; see Table 2 for full results).

Based on the AIC values, the two-part model always provided a better fit than the standard mixed-effects model. Across all 8 models fitted, the difference in AIC values was at least 74 points in favor of the two-part model.

## Discussion

Lardinois and colleagues (14) previously observed that patients with NAPD reported increased negative affect and psychotic experiences in response to both activity stress and event stress. Moreover, they observed that RCA moderated these relationships, such that high levels of RCA were related to more intense emotional and psychotic reactivity to stress. The current study did not unequivocally replicate these findings. While the first analysis, mirroring the statistical approach of the original study, did replicate most findings — the main effects as well as most of the moderation effects — the two-component analysis failed to replicate the moderation effects. Main effects of stress on negative affect and psychotic experiences were not replicated in the mixed-effects logistic regression, but were replicated in the mixed-effects gamma-regression.

Methodological differences between these studies may account for some of the divergence. First, the current study used the CECA, which is an interview-based measure, while the original study used the CTQ, a questionnaire. This may have influenced participant response. Moreover, the CECA does not include a subscale for physical and emotional neglect. Perhaps these subscales accounted for some amount of the original effect. We also observed that more participants failed to fill out the required number of questionnaires (*n* = 21), compared to the original study (*n* = 3). A difference in the workload between the two studies is likely to account for this: the current study is part of a larger project, involving significantly more testing than the original study (see Weijers et al. 22 for the tests used). One could argue that such drop-out may have been selective, thus influencing results. However, *post-hoc* logistic regression revealed that neither severity of psychotic symptoms as measured by the Positive and Negative Syndrome Scale (33) nor RCA predicted dropout from the ESM study (*p*'s > 0.18).

Another possible limitation is that our sample is fairly heterogeneous, including patients with a first-episode psychosis as well as long-term treated patients. Future research concerning stress reactivity in NAPD should attend to these different subtypes and to illness duration. A recent study has shown that age at illness onset has a significant impact on some of the outcomes of schizophrenia (34), which also may have impacted the current results. Also, stress may have divergent effects on different subtypes of NAPD. However, we feel that it is beyond the scope of the current study to examine the impact of these factors; its main aim was to replicate the study by Lardinois et al. (14) and the amount of heterogeneity of both studies is fairly similar. Patients in Lardinois et al.'s study received between 1 month and 10 years of treatment prior to the study, and were between 17 and 45 years old. In our study patients received between 6 months and 10 years of prior treatment, and were between 19 and 57 years old.

Despite the observed differences, we would also like to underscore that we did replicate findings using a similar statistical approach. Even main and interaction effect-sizes were roughly replicated, suggesting that the two studies are not that dissimilar. In our view, the main question here is why there is a lack of statistical support for main effects in the mixed-effects logistic regression as opposed to the mixed-effects gamma regression and why there is a lack of evidence for moderation in the entire two-component analysis as opposed to the standard mixed-effects model. First, the *absence* of statistical evidence for main effects in the mixed-effects logistic regression and the *presence* of statistical evidence in the mixed-effects gamma regression may indicate that stress rather influences the intensity of psychotic symptoms and negative affect than their actual development. Second, concerning the lack of statistical evidence for the moderation effects in the entire two-component analysis, one could argue that the results of the two-component analyses may reflect a lack of power. Out of necessity, to analyse the clumped and right-skewed data, we dichotomized the outcome for the binary logistic analyses. For the gamma models, only data with psychotic experiences or negative affect actually being present was taken into consideration. By breaking down the data in this manner, we may have lost information and therefore power. More participants may be needed to find existing effects with such an approach.

Lastly, a more straightforward interpretation of the data is that such moderation may not actually exist. Stress sensitization is perhaps an incomplete or oversimplified account of the effect of RCA on psychotic symptoms. Perhaps other factors, such as genetic liability (35), outsider status (36), or insecure attachment style (37) may ultimately determine whether abusive experiences in childhood result in psychosis. The significant moderation effects of our primary analysis then may reflect Type I errors caused by violations of assumptions of linearity and normality, which may also account for previous contradictory findings (14, 15). Of course this does not necessarily mean that the original findings (14) are reflective of Type I error as well. However, given that in the original study negative affect and psychotic experiences averaged 1.80 and 1.51 respectively on a scale of 1 to 7—suggestive of heavy right-skewness—a mixed-effects linear regression is unlikely to have been an optimal fit for the data. In defense of the original study, though, we should mention that the analysis of multilevel data is constantly in development. Accounting for violation of normality in hierarchically structured data has only very recently been brought to attention (17) and the violation is complex to address.

In conclusion, the current findings replicated some but not all findings by Lardinois et al. (14). The relationship between stress and the intensity of negative affect/psychotic experiences (when present) seems robust, but the moderating effects of RCA on stress reactivity less so. By using two statistical approaches, this study has furthermore illustrated the importance of exploring the effects of assumption violation on multilevel data. Statistical assumptions may not always be heeded, but can clearly lead to differing conclusions.

We think that the findings of the present study are of clinical relevance. Multiple authors have argued that it may be important to tailor NAPD interventions toward the observed heightened emotional reactivity to daily stressors (12, 13, 15). Indeed, stress seems robustly related to the severity of psychotic experiences and negative affect, at least if already present. This may mean that interventions targeting reactivity to stress are of benefit to patients with NAPD. Interventions such as mentalization based treatment, which is aimed at improving emotion regulation, may be fruitful in this regard (38). However, the current results also increase uncertainty about the pathogenic model put forth by previous articles. There is still uncertainty about whether the relation between stress and the severity of psychotic experiences and negative affect can actually be attributed to childhood abuse. Therefore, interventions aimed at reducing stress reactivity may not affect psychotic experiences attributable to childhood abuse. In our view, it is important to ascertain whether previous results do not reflect Type I errors. Previous findings involving standard mixed-effects models with similar outcomes may need re-analysis. Lastly, future research should determine whether such assumption violations actually lead to different interpretations in sufficiently large samples.

## Ethics statement

Research was conducted according with the provisions of the World Medical Association Declaration of Helsinki. This study is part of a larger study for which ethical approval was given by the Medical Research Ethics Committee of the University Hospital Maastricht and Maastricht University (13-3-066.5/ab). Written informed consent was obtained from all subjects.

## Author contributions

JW developed the study concept and wrote the first draft. JW was also responsible for testing and data collection. WV and JW performed data analysis. WV, EE-B, and J-PS provided critical revisions. All authors approve of the final version of the paper for submission.

### Conflict of interest statement

The authors declare that the research was conducted in the absence of any commercial or financial relationships that could be construed as a potential conflict of interest.
